# Supplementary material for: Activation of PKA via asymmetric allosteric coupling of structurally conserved cyclic nucleotide binding domains
Source: Nat Commun. 2019 Sep 4;10:3984. doi: 10.1038/s41467-019-11930-2 (PMC6726620; doi:10.1038/s41467-019-11930-2)
Supplement: Supplementary file 1 — Supplementary Information [file 41467_2019_11930_MOESM1_ESM.pdf]

SUPPLEMENTARY INFORMATION

FOR

Activation of PKA Via Asymmetric Allosteric Coupling of Structurally Conserved Cyclic Nucleotide Binding Domains

By

Hao *et al.*

SUPPLEMENTARY METHODS

1. Quantitative Analysis of Unfolding and Refolding Force Distribution

The force dependent lifetimes were determined from the unfolding force probability histograms using the method from Dudko *et al*[1,2]. Equation (1) is applied considering an unfolding force histogram containing N bins of the width ΔF that starts at F_o and ends at $F_N = F_0 + N\Delta F$. Let the number of counts in the ith bin be C_i, resulting in a height $h_i = C_i/(N_{tot}\Delta F)$ with N_{tot} equal to the total number of counts and where k = 1, 2,…

$$\tau(F_0 + (k - 1/2)\Delta F) = \frac{(h_k/2 + \sum_{i=k+1}^{N} h_i)\Delta \mathrm{F}}{h_k\dot{F} + (F_{0\_}(k-1/2)\Delta F} \qquad \text{Equation (1)}$$

where $\dot{F}$ is the force loading rate and τ is the force dependent lifetime. In order to extract the force dependent lifetimes from the folding force histograms, we used the following equation[3]:

$$\tau(\mathrm{F}) = \tau_0\left(1 - \frac{vF\Delta x^{\ddagger}}{\Delta G^{\ddagger}}\right)^{1-1/v} e^{-k_B T\Delta G^{\ddagger}\left[1-\left(1-vF\Delta x^{\ddagger}/\Delta G^{\ddagger}\right)^{1/v}\right]} \qquad \text{Equation (2)}$$

where τ_o represents the folded lifetime of the protein in the absence of force; F is the force; $\Delta x^{\ddagger}$ is the distance to the transition state from the folded to the unfolded state. k_B is the Boltzmann constant; and T is the absolute temperature. $\Delta G^{\ddagger}$ is the free-energy of activation in the absence of external force. ν is the scaling factor that specifies the nature of the underlying free-energy landscape. Since the lifetime showed a liner behavior where ν = 1, the equation (1) was simplified to Bell's model. The unfolding force probability distribution was fitted using the same fitting parameter (τ_o and $\Delta x^{\ddagger}$) to the following equation[2]:

$$p(\mathrm{F}) = (\mathrm{KV})^{-1}k(F)e^{k_0/\Delta x^{\ddagger}KV}\times e^{-[\frac{k(F)}{\Delta x^{\ddagger}KV}][1-(\frac{vF\Delta x^{\ddagger}}{\Delta G^{\ddagger}})^{1-\frac{1}{v}}]} \qquad \text{Equation (3)}$$

where KV is the force loading rate, k_0 is the rate of unfolding at zero force, and k(F) is equal to:

$$k(\mathrm{F}) = k_0(1-\frac{vF\Delta x^{\ddagger}}{\Delta G^{\ddagger}})^{\frac{1}{v}-1}\times e^{\Delta G^{\ddagger}[1-\left(1-\frac{vF\Delta x^{\ddagger}}{\Delta G^{\ddagger}}\right)^{\frac{1}{v}}]} \qquad \text{Equation (4)}$$

2. Worm-like chain (WLC) Analysis for isolated CNB Domains.

The extension of the unfolding rip seen in force-extension curves can be analyzed with the Worm-like chain (WLC) model[4] to determine the number of participating amino acids in the unfolding reaction. The WLC equation describes the dependence of force on the molecular extension of a flexible polymer. The resulting force is given by:

$$\mathrm{F} = \frac{k_B T}{p}[\frac{1}{4}\left(1-\frac{x}{L_C}\right)^{-2} - \frac{1}{4} + \frac{x}{L_C}] \qquad \text{Equation (5)}$$

where p is the persistence length of the chain (p = 0.65nm for polypeptides), x is the end-to-end extension, and Lc is the contour length (calculated by multiplying the number of amino acids by 0.365 nm per amino acid). In calculating changes in contour length:

$$\Delta\mathrm{Lc} = \mathrm{Lc} - d_{folded} \qquad \text{Equation (6)}$$

where Lc is the contour length, and d_{folded} is the end-to-end length between the attachment points in the folded protein (determined from the high-resolution structure).

Therefore, it is possible to compare the number of amino acids that encompass each unfolding reaction under different pulling geometries. By using the WLC model, the truncated CNB-A domain (residues 110-243) has an expected change in contour length of

(ΔLc) of 42.8 nm (124 residues × 0.365 nm per residue – 1.89 nm (the distance between residue 110 and 243 in the folded protein)). Since the position 120 is the first structured residue in the CNB-A domain, the total unfolded residue number is 243 – 120 + 1 = 124 instead of 134. In the case of the truncated CNB-B domain (residues 243-376), the expected ΔLc = 48.0nm (134 residues × 0.365 nm per residue – 3.26 nm (the distance between residue 243 and 376 in the folded protein, holoenzyme structure, PDB code: 2QCS[5]).

3. Implementation of Monte-Carlo Simulation

The dynamic trajectory of a single tether was carried out by stochastic Monte-Carlo simulations[6,7]. Briefly, simulations were performed by discretization of simulation time into small units Δt, such that transition probabilities within a given time step were < 0.05. For our simulations, Δt was chosen to be 5 ms, therefore our simulation was sampled at 200 Hz. Within each time step, iteration of the following processes permitted physical simulation of polymer unfolding:

(1) *Calculation of force-extension for worm-like chains in series*: For each polymer unit in the tension chain (i.e. DNA and protein), a force-extension curve is calculated to relate the polymer unit's fractional extension to applied forces between 0 and 20 pN. At each force, the total extension of the tension chain is the sum of the products of fractional extension and contour length for each polymer unit within the tension chain. Parameters for worm-like chain calculations are provided in a separate paragraph below.

(2) *Stretching of worm-like chains in series*: At each time step, the tension force exerted by the polymer chain is balanced by the pulling force exerted by the optical trap plus a random fluctuating force ($\vec{F}$): $\vec{F}_{\text{chain}}(t) + \vec{F}_{\text{trap}}(t) + \vec{\xi}(t) = 0$, where *Δ(t)* is a random number chosen from a zero-mean normal distribution with standard deviation $\sigma = \sqrt{2\, k_{\text{B}} T\, \gamma_0 / \Delta t}$. The Stokes' drag coefficient, $\gamma_0 = 6\pi r \eta$, was calculated as a spherical 2.1–μm diameter bead with radius, $r = 1.05\ \ \mu$m, in a medium with dynamic viscosity, $\eta = 1$ cP. The solution to the above force equation was solved numerically at each time step, which also by extension directly calculated the total extension of tension chain and position of the bead in the trap.

(3) *Protein unfolding transition probabilities*: If protein unit A in the tension chain is folded, it is converted to an unfolded state with probability $P(A) = k_A \exp\left[F(t)\Delta x_A^{\ddagger} / k_B T\right]$. Similarly, if protein unit B in the tension chain is folded, it is converted to an unfolded state with probability $P(B) = k_B \exp\left[F(t)\Delta x_B^{\ddagger} / k_B T\right]$.

(4) *Movement of the trapped bead*: The trapped bead is held in an optical trap with Hookean spring constant, $\kappa = 0.075$ pN/nm. Throughout the simulation, the trap position is moved at a rate of $v = 75$ nm/s, therefore at each time step, the trap position is incremented by $x(t) = x(t-1) + v\Delta t$.

(5) *Time evolution*: Simulation time *t* was incremented by Δt.

(6) *Worm-like chain and other parameters used in simulations*. Worm-like chain and parameters used in simulations: 700 bp of DNA was simulated with a persistence length of P_{DNA} = 50 nm, and the unfolded domains with contour lengths L(length $\Delta Lc_{\text{CNB-A}}$) =

46 nm and L(ΔLc $_{CNB-B}$) = 52 nm for the CNB-A and CNB-B domains, respectively, had a persistence length of P_{unf} = 0.65 nm. The zero-force protein lifetime for 1,600 s for CNB-A domain, and 1,100 s for the CNB-B domain, leading to zero-force unfolding rates of $k_A = 6.25\times10^{-4}$ s^{-1}, and $k_B = 9.09\times10^{-4}$ s^{-1}. The distances to the unfolding transition states used were $\Delta x_A^{\ddagger} = 4.0$ nm, and $\Delta x_B^{\ddagger} = 4.8$ nm. See **Supplementary Table 1** for details.

Discrete time Monte-Carlo simulations were repeated for 2,000 replicates in MATLAB (MathWorks, Natick, Massachusetts), and the features from the resulting stochastic trajectories were plotted directly.

4. Assigning the Structures of the Unfolding Intermediates in Type-III Constructs Regulatory Subunit Bound to cAMP

We use the WLC model[4] to generate a force versus extension upon unfolding plot from type-III (S110C/S376C) trajectories (**Supplementary Fig. 3**). Three WLC curves were generated using a ΔLc of 13 nm (1st rip), 50 nm (2nd rip) and 31 nm (3rd rip). A similar WLC analysis of the truncated CNB-A and CNB-B domains (type I constructs) bound to cAMP matched the 3rd and 2nd larger rips observed in the type-III S110C/S376C regulatory subunit, respectively. This result indicates that the 2nd rip is due to the unfolding of CNB-B domain while the 3rd rip is originated from the CNB-A domain. Based on the comparison between the type-III S110C/S376C and D149C/S376C constructs (**Supplementary Fig. 3**), the 1st rip corresponds to the N3A motif. The CNB-B domain as part of the regulatory

subunit has a longer ΔLc (50 nm) compared to its truncated counterpart (47 nm), likely due to the simultaneous unfolding the CNB-B domain and the B/C helix, which altogether incorporates residues 232 to 376. The distribution of measured contour lengths for the CNB-B domain construct either as a truncation or as part of the regulatory subunit have the expected contour length.

We sought to refine the identity of the secondary structures associated with each unfolding intermediate (or unfolding rip) in the type-III S110C/S376C regulatory subunit. Based on the cAMP-bound high-resolution structure of regulatory subunit (PDB: 1RGS[8]), we mapped the observed rip for each unfolding intermediate to a particular secondary structure using the following equation[9,10]:

$$\Delta L_C = (n+1)\times L_{aa} - (X^D_{m:N\to C} - X^D_{m+n:N\to C}) \qquad \text{Equation (7)}$$

ΔL_c describes the actual change in contour length of each unfolding intermediate (in nm). *n* is the number of residues involved during the transition and L_{aa} is the contour length increment per amino acid (0.365nm/aa). The second term in Equation (7) is the shift in the distance between the last structured residue at the N-terminus (residue position "*m*") and the last structured residue after the unfolding event occurs (residue position "*m*+*n*"). *X* is the folded distance (*D*) from N-terminus to C-terminus (N → C) accounting from residue number *m* or *m*+*n*, which are determined from the crystal structure.

The positions giving the lowest root-mean-square-deviation (RMSD) values from the WLC fit (**Supplementary Fig. 3g, star**) are the optimal residues incorporated in each unfolding intermediate. The 1st rip corresponds to residues 110 to 149, the 2nd rip

corresponds to residues 233 to 376, and the 3rd rip corresponds to residues 150 to 232. These assignments corroborate the experimental comparisons among the truncated CNB domains, and the type-III constructs D149C/S376C and S110C/S376C. Each unfolding intermediate was map onto a topological representation of the regulatory subunit topology (**Supplementary Fig. 3h**).

5. Contribution of the N3A motif to the mechanical stabilization of the CNB-B domain

After the N3A motif is fully unraveled, the CNB-B domain unfolds at F_{avg} = 15.0 ± 0.1 pN (**Fig. 3a, Supplementary Fig. 6c, d** and **Supplementary Table 4**), which is ~ 4 pN lower than the force obtained when the CNB-B domain is selectively unfolded using a type-II construct (**Fig. 2a** and **Supplementary Table 2**). Thus, of the ~ 7 pN of mechanical stabilization conferred by the CNB-A domain, the N3A motif alone contributes 4 pN. The other 3 pN reflect partial stabilization of inter-domain interactions, likely mediated by W260 located in the CNB-B domain that serves as adenine capping residue of the cAMP bound to the CNB-A domain[12,20]. The last step in the unfolding pathway of S110C/S376C corresponds to the CNB-A domain, which occurs at forces similar to those obtained for the isolated domain since other interacting domains are no longer folded (**Fig. 3a** and **Supplementary Fig. 6c, d**).

6. Contact map analysis for PKA regulatory subunit

Pairwise contact map comparing the interaction established by the N3A motif in the regulatory subunit for wild type and R241A was analyzed with CMView software[11]. The PDB code used for PKA regulatory subunit is 1RGS. The atomic coordinates of the simulated R241A structure was provided by Emília Pécora de Barros from the Amaro lab at UCSD. The contact map is based on Cα atoms using a cutoff distance of 8 Å. The CMView software can be freely obtained at:

http://www.bioinformatics.org/cmview/installation.html

7. cAMP Titration of Type-I and Type-III constructs

Type-I isolated CNB-A and Type-I isolated CNB-B constructs were used in the cAMP titration experiments to determine the binding affinity of each domain. By counting the fraction of bound and unbound at different cAMP concentration, we were able to build a single-molecule titration curve for each isolated domain. The binding affinity of each domain was calculated using the following equation:

$$\text{Fraction Bound} = \frac{k \cdot [cAMP]}{1 + k \cdot [cAMP]} \quad \text{Equation (8)}$$

We obtained $k_{\text{CNB-A}} = 1.2 \pm 0.4 \cdot 10^7$ M and $k_{\text{CNB-B}} = 3.7 \pm 1.0 \cdot 10^7$ M for type-I isolated CNB-A and CNB-B respectively (**Supplementary Fig. 5**).

Type-III Regulatory subunit S110C/S376C was used in the cAMP titration experiments (**Fig. 5c**). At different cAMP concentration, every trajectory was assigned to apo (A_0B_0), partial liganded state (A_1B_0 or A_0B_1) or fully bound (A_1B_1). The assignment was based on

the force and changes in extension (ΔLc) signatures from experiments with type-I and type-III constructs in the apo state or saturated with cAMP. The CNB domains in the apo state always unfold at forces below 10 pN and with ΔLc between 45 nm and 49 nm. Therefore, during the titration of cAMP, two consecutive unfolding rips that occurred at forces < 10 pN with 45 nm < ΔLc < 49 nm were assigned to the apo state. Trajectories with three unfolding rips with ΔLc = 13 nm for the 1st rip, 50 nm for the 2nd rip, and 31 nm for the 3rd rip (like the ones obtained using saturating amounts of cAMP, 0.5 mM) were assigned to the fully bound state. Trajectories with one rip with an unfolding force and ΔLc similar to that of the isolated cAMP-bound CNB-A domain (10-20 pN, ΔLc = 31 nm) and one rip occurring at a lower force (5-12pN) was assigned to A_1B_0. The rest of trajectories were assigned to A_0B_1. A titration curve of each liganded state was plotted, with the error bar showing the weighted standard deviation of different molecules.

We established a binding model between cAMP and the two binding sites of the regulatory subunit:

$$\begin{array}{ccc} A_0B_0 + \text{cAMP} & \overset{k_1}{\leftrightarrows} & A_{\text{cAMP}}B_0 + \text{cAMP} \\ k_2 \downarrow\uparrow & & \downarrow\uparrow k_3 \\ A_0B_{\text{cAMP}} + \text{cAMP} & \underset{k_4}{\leftrightarrows} & A_{\text{cAMP}}B_{\text{cAMP}} \end{array}$$

Each liganded state fraction as a function of cAMP concentration is describe using the following equations:

$A_0B_0 = 1/ (1 + k_1\cdot[cAMP]+ k_2\cdot[cAMP]+ k_1\cdot k_3\cdot[cAMP]^2)$

$A_1B_0 = k_1\cdot[cAMP]/ (1 + k_1\cdot[cAMP]+ k_2\cdot[cAMP]+ k_1\cdot k_3\cdot[cAMP]^2)$

$A_0B_1 = k_2\cdot[cAMP]/ (1 + k_1\cdot[cAMP]+ k_2\cdot[cAMP]+ k_1\cdot k_3\cdot[cAMP]^2)$

$A_1B_1 = k_1\cdot k_3\cdot[cAMP]^2/ (1 + k_1\cdot[cAMP]+ k_2\cdot[cAMP]+ k_1\cdot k_3\cdot[cAMP]^2)$

We used *PyFolding* software to proceed the global fitting of four liganded state curves with shared parameters, where $k_1 = 5.7 \pm 0.5\cdot10^7$ M^{-1}, $k_2 = 1.0 \pm 0.1\cdot10^8$ M^{-1}, $k_3 = 1.5 \pm 0.2\cdot10^8$ M^{-1} and $k_4 = 8.7 \pm 1.2\cdot10^7$ M^{-1}. The *PyFolding* Script can be freely obtained at: https://github.com/quantumjot/PyFolding[12]

8. Bayesian Hidden Markov Model (BHMM) analysis

We used a Bayesian Hidden Markov Model (BHMM) approach to analyze the optical tweezers data collected under force-clamp experiments[13]. The BHMM analysis method has been previously described and applied in analyzing single molecule trajectories[14]. The MATLAB code used for the BHMM approach can be freely obtained at: http://simtk.org/home/bhmm.

9. Calculation of Equilibrium Free Energy

Having obtained the lifetimes of the folded ($\tau_{0,F}$) and unfolded states ($\tau_{0,U}$) at zero force, we estimated the equilibrium free energy of unfolding of each CNB domain using the following equation:

$$\Delta G^0 = -RTln\frac{k_{F\to U}}{k_{U\to F}} = -RTln\frac{\tau_{0,U}}{\tau_{0,F}} \quad \text{Equation (9)}$$

where RT = 0.592 kcal/mol. The calculations were done with Type-I and Type-II constructs, with or without cAMP. For the CNB-A domain bound to cAMP, $\tau_{0,F}$ was obtained after the N3A motif had unfolded (ΔLc = 30.2 nm), whereas $\tau_{0,U}$ was obtained for the full-length protein (ΔLc = 43.1 nm). Because these two lifetimes represent different kinetic steps in the unfolding and refolding reactions, and in particular the unfolding reaction does not consider the energy required to unfold the N3A motif, we can only place a lower boundary of ΔG^0 for the cAMP-bound CNB-A domain either as a truncation (Type-I construct) or as part of the PKA regulatory subunit (Type-II construct)(**Supplementary Table 2**). For other conditions (i.e., apo state) or for the CNB-B domain, the folded and unfolded state lifetimes represented the same kinetic step and therefore equation 5 can be directly applied.

10. Calculation the Change of Solvent Accessible Surface Areas

The solvent accessible surface areas (ASA) of regulatory subunit bound with cAMP (PDB: 1RGS) and bound with the catalytic subunit (PBD: 2QCS) was calculated. The total change of ASA is the sum of the change in ASA for every residue in the regulatory subunit. The online calculation resources can be freely accessed at: http://curie.utmb.edu/getarea.html[15]

Region of the change in ASA	Percentage

N3A motif	24%
CNB-A domain (not including N3A motif)	18%
B/C helix	26%
CNB-B domain	33%

11. Calculation of the Stability of the N3A Motif in the Regulatory Subunit Bound to cAMP (Type-III S110C/S376C construct)

Force-clamp data was used to estimate the stability change of the N3A motif from the folded state (F) to the unfolded state (U). This is accomplished using the following the equation[14]:

$$\Delta G_0 = \Delta G_{work} - \Delta G_{stretch} \qquad \text{Equation (10)}$$

where $\Delta G_{work} = F_{1/2} \times \Delta x_{U\text{-}F}$. $F_{1/2}$ is the force in which the folded and unfolded states are equally populated (equilibrium force), and $\Delta x_{U\text{-}F}$ is the extension change difference between U and F. For the wild type regulatory subunit (Type-III S110C/S376C), $F_{1/2}$ = 11 pN with $\Delta x_{U\text{-}F}$ = 5.5 nm, resulting in ΔG_{work} = 60 pN•nm. Based on the Worm-like chain (WLC) model (SI section 2) and using a persistence length for polypeptides of 0.65 nm, a change in extension of 5.5 nm at 11 pN is equal to a change in contour length of ΔLc = 9.5 nm. $\Delta G_{stretch}$ is the integration of the WLC model using a ΔLc = 9.5 nm with boundaries from F = 0 pN to $F_{1/2}$ = 11 pN, yielding 21.75 pN•nm. Therefore ΔG_0 = (60 – 22) pN•nm = 38 pN•nm = 9.2 k_BT = 5.4 kcal/mol. A similar analysis for the R241A mutation (Type-

III S110C/S376C) yielded ΔG_0 = 4.7 kcal/mol.

SUPPLEMENTARY TABLES

Supplementary Table 1

Kinetic and thermodynamic parameters of type-I (isolated domain) and type-II (selective domain unfolding in PKA regulatory subunit) constructs in the apo state

Construct	Unfolding Force ± std. (pN) (number of events)	Folded State $\tau_{0,F}$ ± std. (s)	$\Delta x^{\ddagger}_{F\rightarrow U}$ ± std. (nm)	Unfolded State $\tau_{0,U}$ ± std. (s)	$\Delta x^{\ddagger}_{U\rightarrow F}$ ± std. (nm)	ΔL_c± std. (nm)	ΔG^0 (kcal/mol)
Type-I: CNB-A	8.8±1.3 (N=1114)	$1.6\pm0.4\cdot10^{3}$	4.0±0.2	$2.1\pm1.4\cdot10^{-4}$	9.2±0.9	43.1±3.3	9.4
Type-I: CNB-B	7.3±1.3 (N=744)	$1.1\pm0.3\cdot10^{3}$	4.8±0.2	$3.6\pm2.3\cdot10^{-3}$	6.9±0.9	50.3±2.7	7.6
Type-II: CNB-A	8.6±1.2 (N=894)	$2.2\pm0.6\cdot10^{3}$	4.2±0.2	$4.3\pm1.0\cdot10^{-4}$	9.7±0.3	44.3±2.9	9.2
Type-II: CNB-B	7.9±1.1 (N=795)	$1.7\pm0.3\cdot10^{3}$	4.3±0.2	$2.6\pm0.4\cdot10^{-3}$	7.4±0.2	49.9±2.8	7.9

Supplementary Table 2

Kinetic and thermodynamic parameters of type-I (isolated domain) and type-II (selective domain unfolding in PKA regulatory subunit) constructs bound to cAMP

Construct	Unfolding Force ± std. (pN) (number of events)	Folded State $\tau_{0,F}$ ± std. (s)	$\Delta x^{\ddagger}_{F\rightarrow U}$ ± std. (nm)	Unfolded State $\tau_{0,U}$ ± std. (s)	$\Delta x^{\ddagger}_{U\rightarrow F}$ ± std. (nm)	ΔL_c ± std. (nm)	ΔG^0 (kcal/mol)
Type-I: CNB-A	17.4±2.0 (N=785)	$1.1\pm0.3\cdot10^{4}$	2.4±0.1	$4.4\pm2.4\cdot10^{-5}$	10.2±0.6	30.2±2.8	>11.5
Type-I: CNB-B	12.0±1.0 (N=608)	$3.9\pm0.7\cdot10^{4}$	4.0±0.1	$1.0\pm0.1\cdot10^{-3}$	8.8±0.2	45.0±2.5	10.4
Type-II: CNB-A	20.3±1.4 (N=1152)	$1.8\pm0.3\cdot10^{6}$	3.2±0.2	$4.1\pm3.0\cdot10^{-6}$	16.9±1.1	30.7±1.4	>15.9
Type-II: CNB-B	19.7±1.6 (N=1518)	$1.4\pm0.5\cdot10^{5}$	2.7±0.1	$1.5\pm1.0\cdot10^{-5}$	13.5±0.9	47.0±1.9	13.6

Supplementary Table 3

Kinetic parameters of type-III constructs (unfolding both domains simultaneously) in intermediate-liganded states

Construct*	Unfolding Force ± std. (pN) (number of events)	Folded State $\tau_{0,F}$ ± std. (s)	$\Delta x^{\ddagger}_{F\rightarrow U}$ ± std. (nm)	ΔL_c ± std. (nm)
Type-III: CNB-A (A_1B_0)	15.7±2.0 (N=285)	$1.0\pm0.4\cdot10^4$	2.7±0.3	31.3±2.5
Type-III: CNB-B (A_1B_0)	10.0±1.4 (N=285)	$4.0\pm2.0\cdot10^3$	4.6±0.3	49.4±2.7
Type-III: CNB-A (A_0B_1)	9.7±1.2 (N=195)	$2.8\pm1.9\cdot10^3$	3.8±0.3	44.8±4.8
Type-III: CNB-B (A_0B_1)	12.5±1.0 (N=195)	$5.3\pm0.5\cdot10^4$	3.8±0.1	45.5±4.2

*Subscripts "1" and "0" denote a domain that is cAMP bound or unbound, respectively.

Supplementary Table 4

Kinetic parameters of type-III constructs (unfolding both domains simultaneously) for wild type and R241A in the absence and presence of cAMP

Construct		Unfolding Force ± std. (pN) (number of events)	Folded State $\tau_{0,F}$ ± std. (s)	$\Delta x^{‡}_{F\rightarrow U}$ ± std. (nm)	ΔL_c± std. (nm)
Type-III wild type unbound	CNB-A	8.3±1.4 (N=592)	$1.0{\pm}0.3{\cdot}10^{3}$	4.2±0.2	46.5±2.6
	CNB-B	8.0±1.1(N=592)	$1.6{\pm}0.110^{3}$	4.5±0.1	54.1±2.6
Type-III wild type bound	CNB-A	17.2±2.5 (N=739)	$2.8{\pm}0.5{\cdot}10^{4}$	2.7±0.1	30.4±4.7
	CNB-B	15.0±2.2 (N=739)	$1.7{\pm}0.2{\cdot}10^{5}$	3.7±0.1	49.4±4.0
Type-III R241A unbound	CNB-A	9.0±1.1 (N=480)	$2.0{\pm}0.3{\cdot}10^{3}$	4.0±0.4	46.5±2.5
	CNB-B	8.3±0.8 (N=480)	$2.1{\pm}0.5{\cdot}10^{3}$	4.1±0.2	54.1±2.9
Type-III R241A bound	CNB-A	18.2±1.5 (N=339)	$4.0{\pm}0.3{\cdot}10^{4}$	2.6±0.1	30.6±1.5
	CNB-B	12.7±0.7 (N=339)	$7.1{\pm}1.3{\cdot}10^{4}$	4.1±0.1	49.0±2.4

Supplementary Table 5

Kinetic parameters of type-I (isolated domains) and type-III (unfolding both domains simultaneously) constructs bound to cGMP

Construct	Unfolding Force ± std. (pN) (number of events)	Folded State $\tau_{0,F}$ ± std. (s)	$\Delta x^{\ddagger}_{F\rightarrow U}$ ± std. (nm)	ΔL_c± std. (nm)
Type-I: CNB-A	12.1±1.1 (N=322)	$7.3\pm1.1\cdot10^{3}$	3.5±0.1	36.8±3.1
Type-I: CNB-B	10.2±0.8 (N=427)	$3.4\pm1.8\cdot10^{3}$	3.8±0.2	47.5±2.1
Type-III: CNB-A	14.1±1.1 (N=316)	$1.2\pm0.2\cdot10^{4}$	3.1±0.1	36.1±2.8
Type-III: CNB-B	12.6±0.9 (N=316)	$6.0\pm1.3\cdot10^{4}$	4.0±0.1	51.7±2.7

Supplementary Table 6

Primer sequence for site-directed mutagenesis

CNB-B deletion from RIα (91-379)

Fwd: 5’-P-TGTTATGAGGAGTTTCTTAGTAAAGTGTC-3’

Rev:5’-P-GCCCATATGTATATCTCCTTCTTAAAG -3’

CNB-A deletion from RIα (91-379)

Fwd: 5’-P-TCACTTCCGCTTTCTCAGCGT-3’

Rev: 5’-P-TGACCATGGAATTCGAAGCTTGAT-3’

C345A in RIα (91-379)

Fwd: 5’-GCCCGTGGCCCGCTGAAGGCCGTCAGGC-3

Rev: 5’-CGGCCGGTCCAGCTTGACGGCCTTCAGC-3’

C360A in RIα (91-379)

Fwd: 5’-CGCCTTCTCGGCCCGGCCTCCGACATCC-3’

Rev: 5’-CTTGAGGATGTCGGAGGCCGGGCCGAG-3’

S110C in RIα (91-379)

Fwd: 5’-GGATGCCGCGTGCTATGTTCGGAAGGTT-3’

Rev: 5’-CCGAACATAGCACGCGGCATCCTCCTCG-3’

S376C in RIα (91-379)

Fwd: 5’-GCTTCGTGTGCCTGTCTGTCT-3’

Rev: 5’-GACAGCCAGGCACACGAAGCT-3’

M243C in RIα (91-379)

Fwd: 5'-GGGAAGCACGCTGAGAAAGAGGAAGTG-3'

Rev: 5'-CACTTTACTAAGAAACTCCTCATAACACT-3'

D149C in RIα (91-379)

Fwd:5'-CATTTTTTGCGCCATGTTCCCGGTTTCCTTTATTG-3'

Rev:5'-CATGGCGCAAAAAATGTCACTTCTCTCGTTATCATC-3'

R241A in RIα (91-379)

Fwd 5'-GGGAAGCACGCTGAGAAAGGCGAAGATGTATGAGGAG-3'

Rev 5'-CTCCTCATACATCTTCGCCTTTCTCAGCGTGCTTCCC-3'

Double-stranded Oligo (dsOligo)

Fwd shared by both dsOligos:

5′-Thiol-GTTACGCCTATTCCTATCATATGAAGACAC-3'

Rev for dsOligo 1:

5′-Phosphate- GGAGTGTCTTCATATGATAGGAATAGGCGTAAC-3'

Rev for dsOligo 2:

5′-PhosphateCGACGTGTCTTCATATGATAGGAATAGGCGTAAC-3'

SUPPLEMENTARY REFERENCES

1. Dudko, O. K., Hummer, G. & Szabo, A. Theory, analysis, and interpretation of single-molecule force spectroscopy experiments. *Proc. Natl. Acad. Sci.* **105,** 15755–15760 (2008).
2. Dudko, O. K., Hummer, G. & Szabo, A. Intrinsic rates and activation free energies from single-molecule pulling experiments. *Phys. Rev. Lett.* **96,** (2006).
3. Bell, G. I. Models for the specific adhesion of cells to cells. *Science* **200,** 618–627 (1978).
4. Bustamante, C., Marko, J. F., Siggia, E. D. & Smith, S. Entropic elasticity of lambda-phage DNA. *Science* **265,** 1599–1600 (1994).
5. Kim, C., Cheng, C. Y., Saldanha, S. A. & Taylor, S. S. PKA-I Holoenzyme Structure Reveals a Mechanism for cAMP-Dependent Activation. *Cell* **130,** 1032–1043 (2007).
6. Van Kampen, N. G. Stochastic Processes in Physics and Chemistry. *Stochastic Processes in Physics and Chemistry* 96–133 (2007). doi:10.1016/B978-044452965-7/50016-7
7. England, J. P. *et al.* Switching of the folding-energy landscape governs the allosteric activation of protein kinase A. *Proc. Natl. Acad. Sci.* **115,** E7478–E7485 (2018).
8. Su, Y. *et al.* Regulatory subunit of protein kinase A: structure of deletion mutant with cAMP binding domains. *Science* **269,** 807–813 (1995).

9. Sen, M. *et al.* XThe ClpXP protease unfolds substrates using a constant rate of pulling but different gears. *Cell* **155,** (2013).

10. Ries, J., Kaplan, C., Platonova, E., Eghlidi, H. & Ewers, H. A simple, versatile method for GFP-based super-resolution microscopy via nanobodies. *Nat. Methods* **9,** 582–584 (2012).

11. Vehlow, C. *et al.* CMView: Interactive contact map visualization and analysis. *Bioinformatics* **27,** 1573–1574 (2011).

12. Lowe, A. R., Perez-Riba, A., Itzhaki, L. S. & Main, E. R. G. PyFolding: Open-Source Graphing, Simulation, and Analysis of the Biophysical Properties of Proteins. *Biophys. J.* **114,** 516–521 (2018).

13. Robert, C. P., Celeux, G. & Diebolt, J. Bayesian estimation of hidden Markov chains: a stochastic implementation. *Stat. Probab. Lett.* **16,** 77–83 (1993).

14. Kaiser, C. M., Goldman, D. H. & Chodera, J. D. Nascent Protein Folding. 1723–1728 (2011).

15. Fraczkiewicz, R. & Braun, W. Exact and efficient analytical calculation of the accessible surface areas and their gradients for macromolecules. *J. Comput. Chem.* **19,** 319–333 (1998).

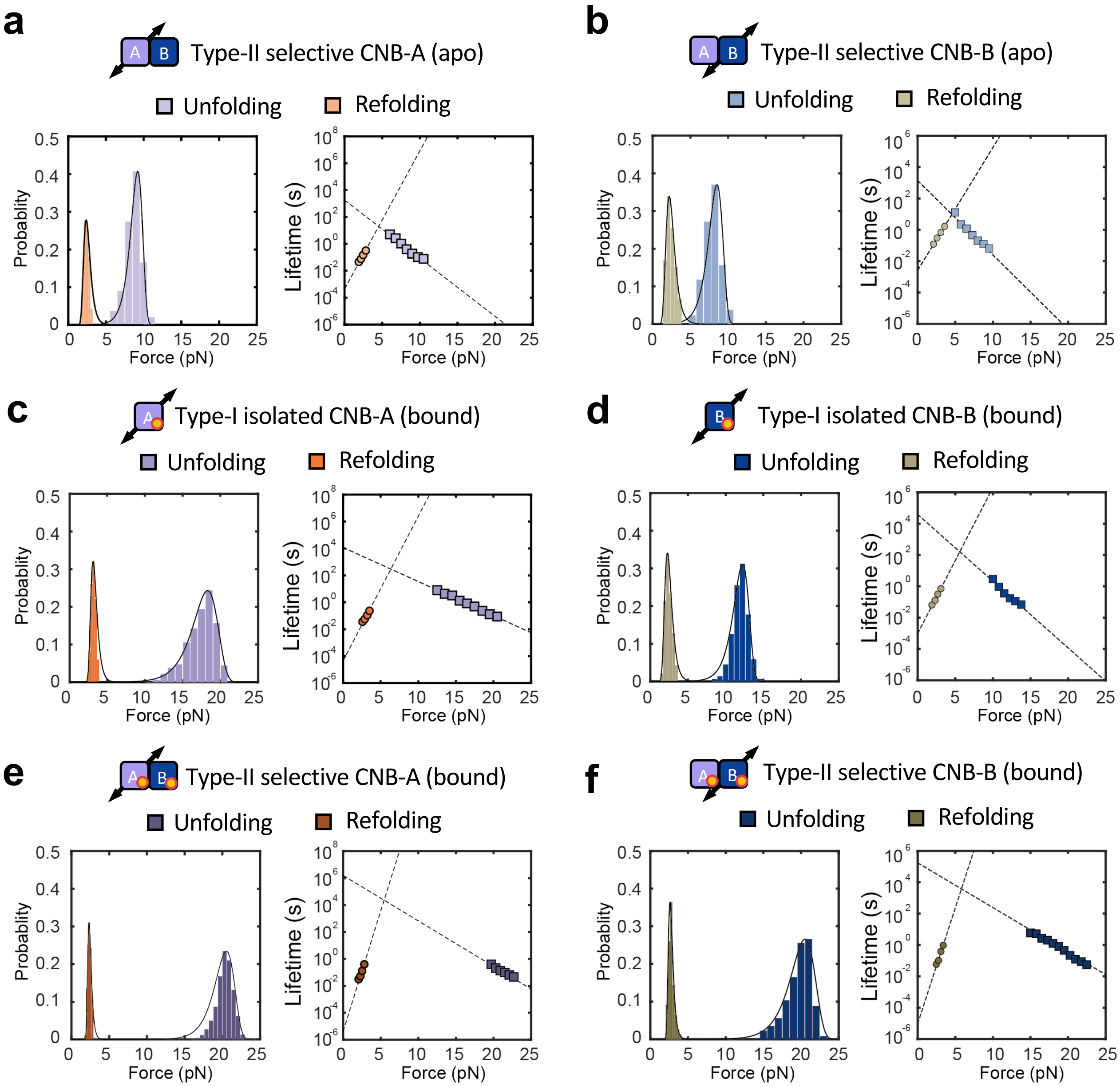

Supplementary Figure 1 | Unfolding and refolding force probability distributions and associated force-dependent folded and unfolded state lifetime with and without cAMP. **a,** CNB-A domain in type-II construct in apo state **b,** CNB-B domain in type-II construct in apo state. **c,** CNB-A domain in type-I construct in cAMP-bound state. **d,** CNB-B domain in type-I construct in cAMP-bound state. **e,** CNB-A domain in type-II construct in cAMP-bound state. **f,** CNB-B domain in type-II construct in cAMP-bound state. Source data are provided as a Source Data file.

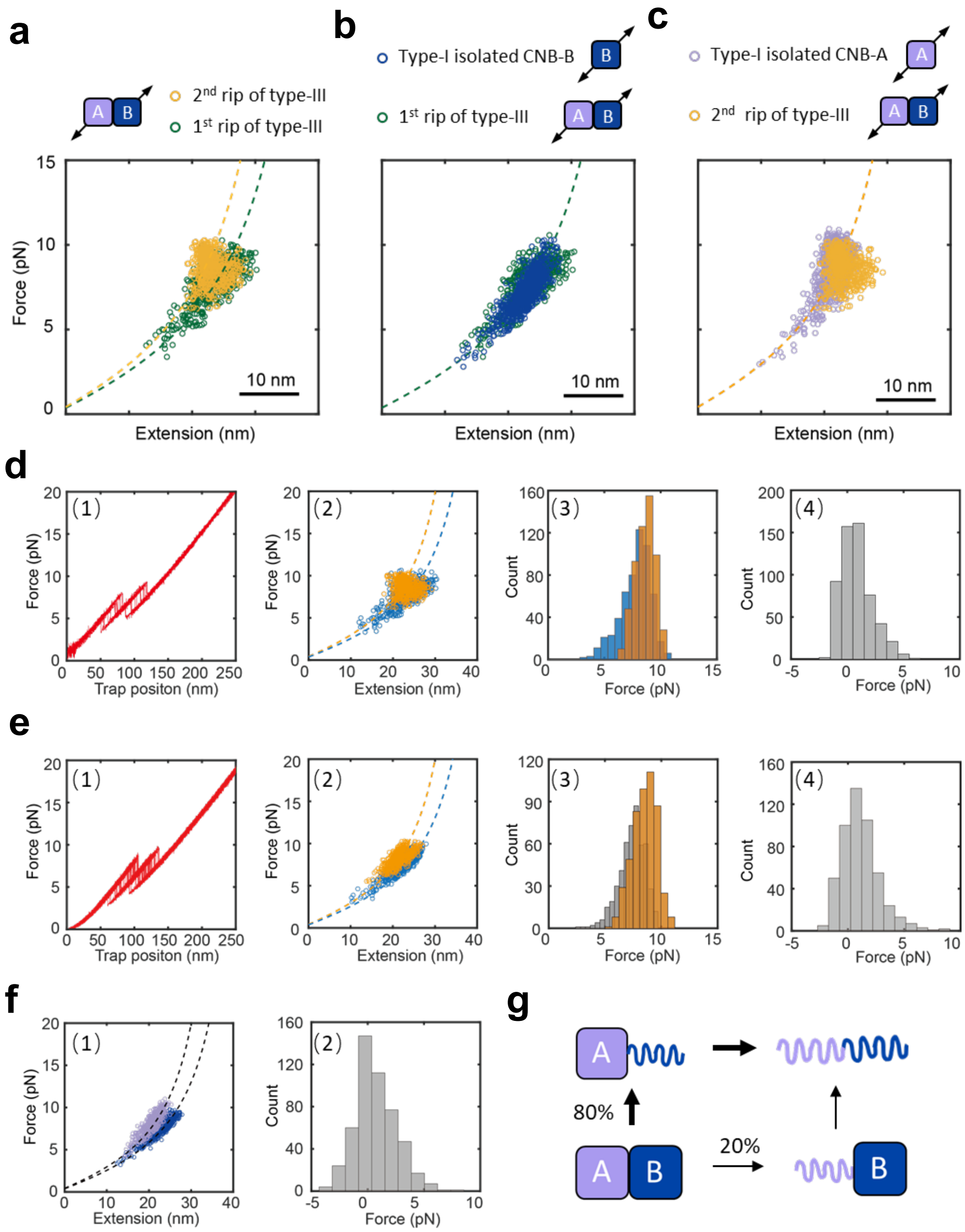

Supplementary Figure 2 | WLC analysis and Monte-Carlo simulations of type-III (S110C/S376C) and type-I constructs. a, Change in extension upon unfolding vs. force of the two rips observed in the type-III construct. The dashed lines are the experimentally determined WLC curves for the isolated CNB-A domain (orange, ΔLc = 43 nm) and the CNB-B domain (green, ΔLc = 50 nm). **b,** The 1st unfolding rip in the type-III construct is indistinguishable from those obtained using a type-I CNB-B domain. The dashed line is the experimentally determined WLC curve for the CNB-B domain (green, ΔLc = 50 nm). **c,** The 2nd rip in the type-III construct is indistinguishable from those obtained using the type-I CNB-A domain. The dashed line is the experimentally determined WLC curve for the CNB-A domain (orange, ΔLc = 43 nm). **d,** Experimental data (N=559). (1) Representative force-extension curve obtained with optical tweezers using the type-III construct. (2) WLC analysis of changes in extension upon unfolding vs. force for the 1st (blue) and 2nd rips (orange). The WLC curves were generated using a ΔLc of 43 nm (yellow) and 50 nm (blue) corresponding to the CNB-A and CNB-B domains, respectively (SI Section 7). (3) Unfolding force histogram for the 1st and 2nd rips. (4) Unfolding force difference: 2nd rip minus 1st rip. **e,** Monte-Carlo simulation (N=559). (1) Simulated force-extension curves of the type-III construct were obtained using the kinetic parameters from the individual CNB domains (SI Table 1 and SI Section 8). (2) WLC analysis of the 1st (blue) and 2nd rips (orange) extracted from the simulated force-extension curves. The WLC curves were the same as in 2A. (3) Simulated unfolding force histogram for the 1st and 2nd rips. (4) Simulated unfolding force difference: 2nd rip minus 1st rip. **f,** (1) The Monte-Carlo simulation revealed the identity of the each CNB domain in the plot of changes in extension vs. force. Data for the CNB-B domain is in blue and corresponds to the 1st rip in 80% of all simulated trajectories. Data for the CNB-A domain is in purple. The WLC curves were the same as in 2A. (2) Simulated unfolding force difference between CNB domains: CNB-A minus CNB-B **g,** Cartoon representing the unfolding pathway of the type-III construct reconstructed from the Monte-Carlo simulation. (For detailed analysis, refer to SI Section 2-3)

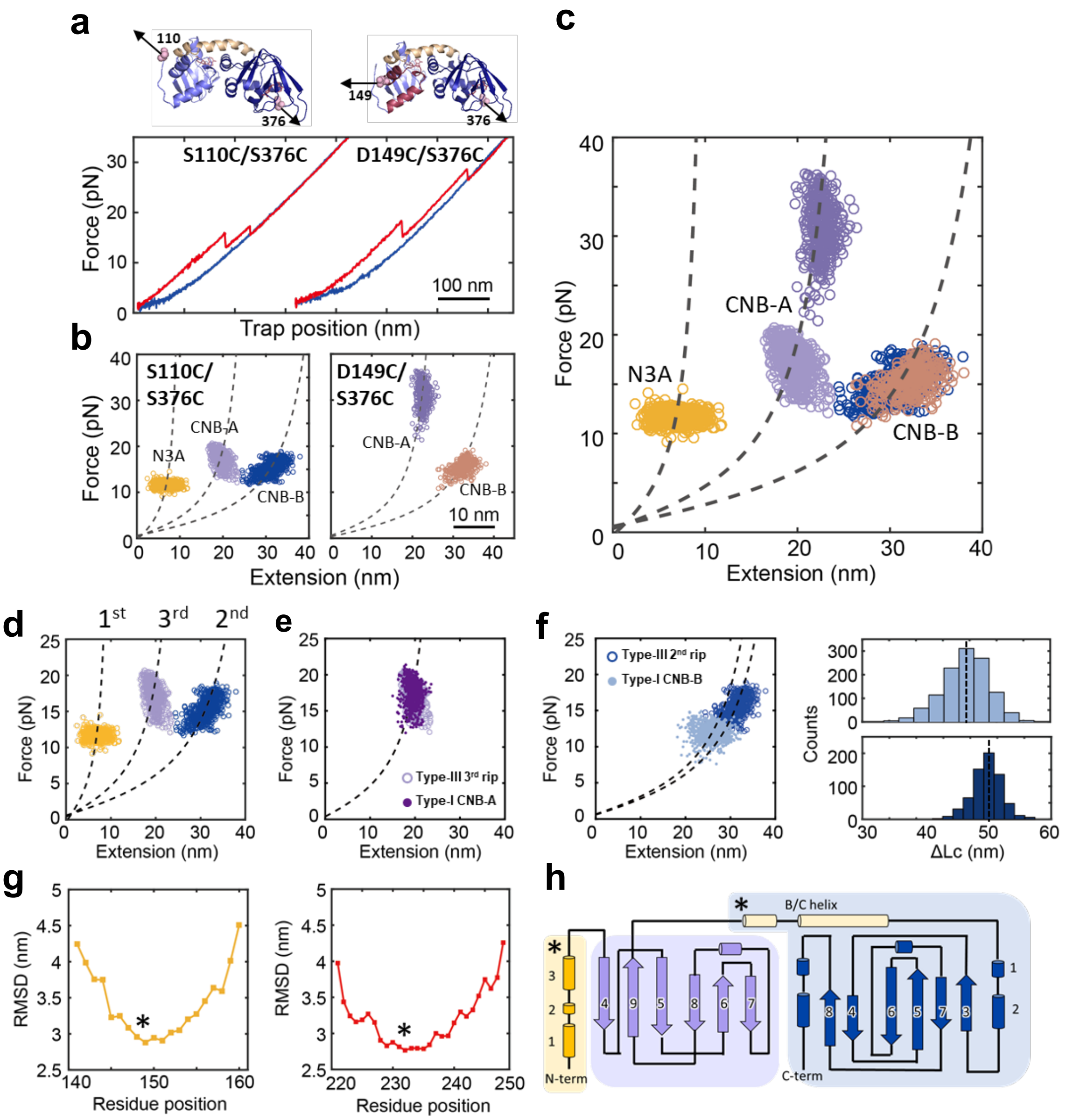

Supplementary Figure 3 | Identification of the N3A motif and assignment of unfolding intermediate for type-III construct in cAMP-bound state. a, Force-extension curve of type-III constructs with DNA handles at residue positions S110C/S376C (left) and D149C/S376C (right). Curves were obtained with cAMP. **b,** WLC analysis of changes in extension upon unfolding vs. force obtained for S110C/S376C (left) and D149C/S376C (right). The WLC curves (dashed lines) were obtained using a ΔLc of 13 nm (for the N3A motif), 31 nm (for the CNB-A domain) and 50 nm (for the CNB-B domain). The D149C/S376C does not show a transition corresponding to the N3A motif. **c,** Combined plot of changes in extension upon unfolding vs. force obtained for S110C/S376C and D149C/S376C. **d,** WLC plot of type-III construct (S110C/S376C) with cAMP. The WLC curves (dashed lines) were obtained using a ΔLc of 13 nm (for the N3A motif, yellow), 31 nm (for the CNB-A domain, purple) and 50 nm (for the CNB-B domain, blue). **e,** WLC analysis (using ΔLc = 31 nm) of the changes in extension upon unfolding for the CNB-A domain in the regulatory subunit (type-III construct) overlaid with the data obtained with the isolated CNB-A domain (type-I construct). **f,** WLC analysis (using ΔLc = 47 and 50 nm) of the changes in extension upon unfolding for the CNB-B domain in the regulatory subunit (type-III construct, dark blue symbols) overlaid with the data obtained with the isolated CNB-B domain (type-I construct, light blue symbols). The CNB-B domain in the regulatory subunit has a longer ΔLc (right panels) likely due to the complete folding of the B/C helix. **g,** Mapping the measured ΔLc for each unfolding rip to the corresponding structural elements of the regulatory subunit. Root-mean-square-deviation (RMSD) of the measured extension changes from the predicted WLC extension change as a function of the residue position for the 1st rip (yellow) and 2nd rip (red). The residue that gives the lowest RMSD is labelled with a star. These residues correspond to 149 (1st rip) and 233 (2nd rip). **h,** Topology of the regulatory subunit showing the identified structural elements in each unfolding rip. The 1st rip corresponds to unfolding of the N3A motif (residues 120-149), the 2nd rip corresponds to the CNB-B domain with the BC helix (residues 233-376), and the 3rd rip corresponds to the CNB-A domain minus the N3A motif (residues 150-233).

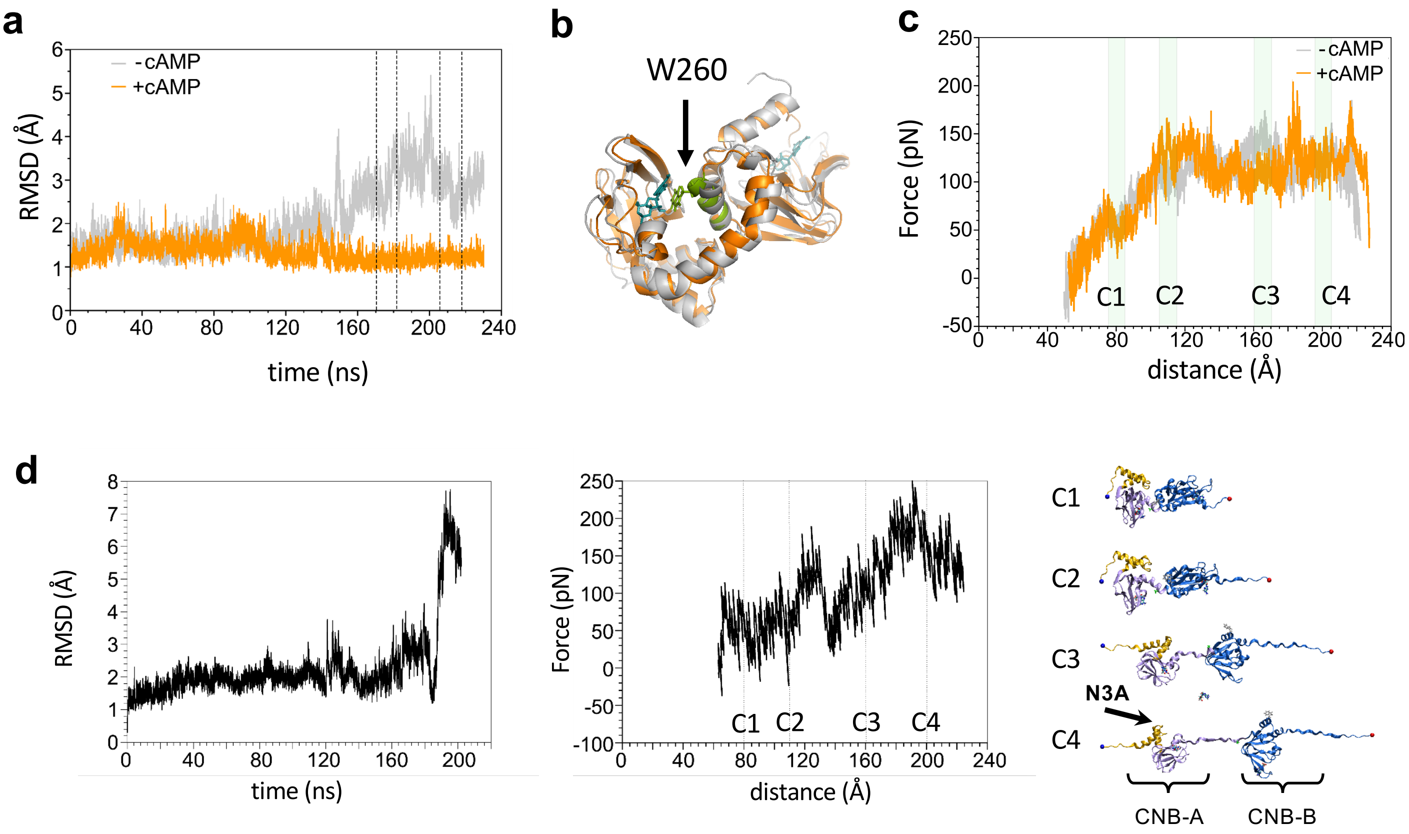

Supplementary Figure 4 | Steered molecular dynamic (SMD) simulations. a, Time series of the RMSD from the crystal structure for the PKA regulatory subunit with (orange) and without cAMP (gray). Vertical dotted lines indicate the frames used as starting points for SMD simulations. **b,** Representative structure of the most populated clusters in the last 50 ns of MD simulations with (orange) and without cAMP (gray); highlighted W260 interacting with cAMP docked in the binding site of the CNB-A domain. In the apo state, the lack of the interaction between cAMP and W260 and the absence of cAMP in the CNB-B binding site promote conformational rearrangement of the αA:B helix (green) causing a reciprocal orientation of the domains. **c.** Force-extension profiles for all the SMD simulations with (orange) and without cAMP (gray). Cluster analysis over selected structures (light green shaded areas) were used to characterize the most probable conformations along the trajectories. Yellow: N3A motif; Purple: CNB-A; Dark blue: CNB-B domain. **d**, RMSD time series (left) for MD, force-extension profile for SMD simulations (middle) and selected conformations (gray dashed lines) along the trajectories (right) for mutant R241A with cAMP along SMD.

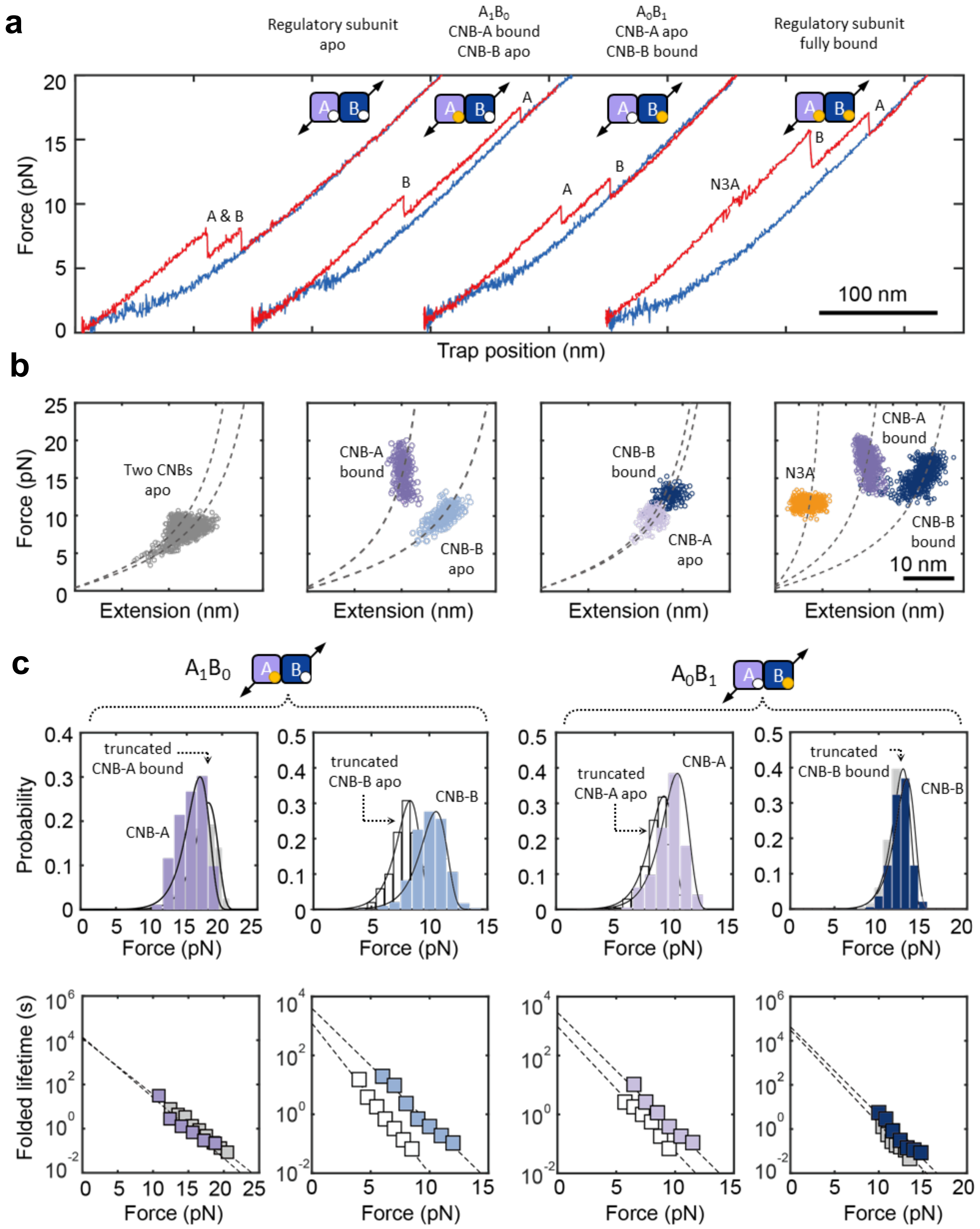

Supplementary Figure 5 | Analysis of intermediate liganded states in cAMP titration of type-III construct. a, Representative force-extension curves of four cAMP-bound states using the type-III construct. **b,** Corresponding WLC analysis of changes in extension upon unfolding vs. force. **c,** Unfolding force probability distributions and force-dependent folded state lifetimes of each CNB domain in two intermediate liganded states. The corresponding truncations in the ligand-free (white) and ligand-bound (gray) state were superimposed for comparison. Source data are provided as a Source Data file.

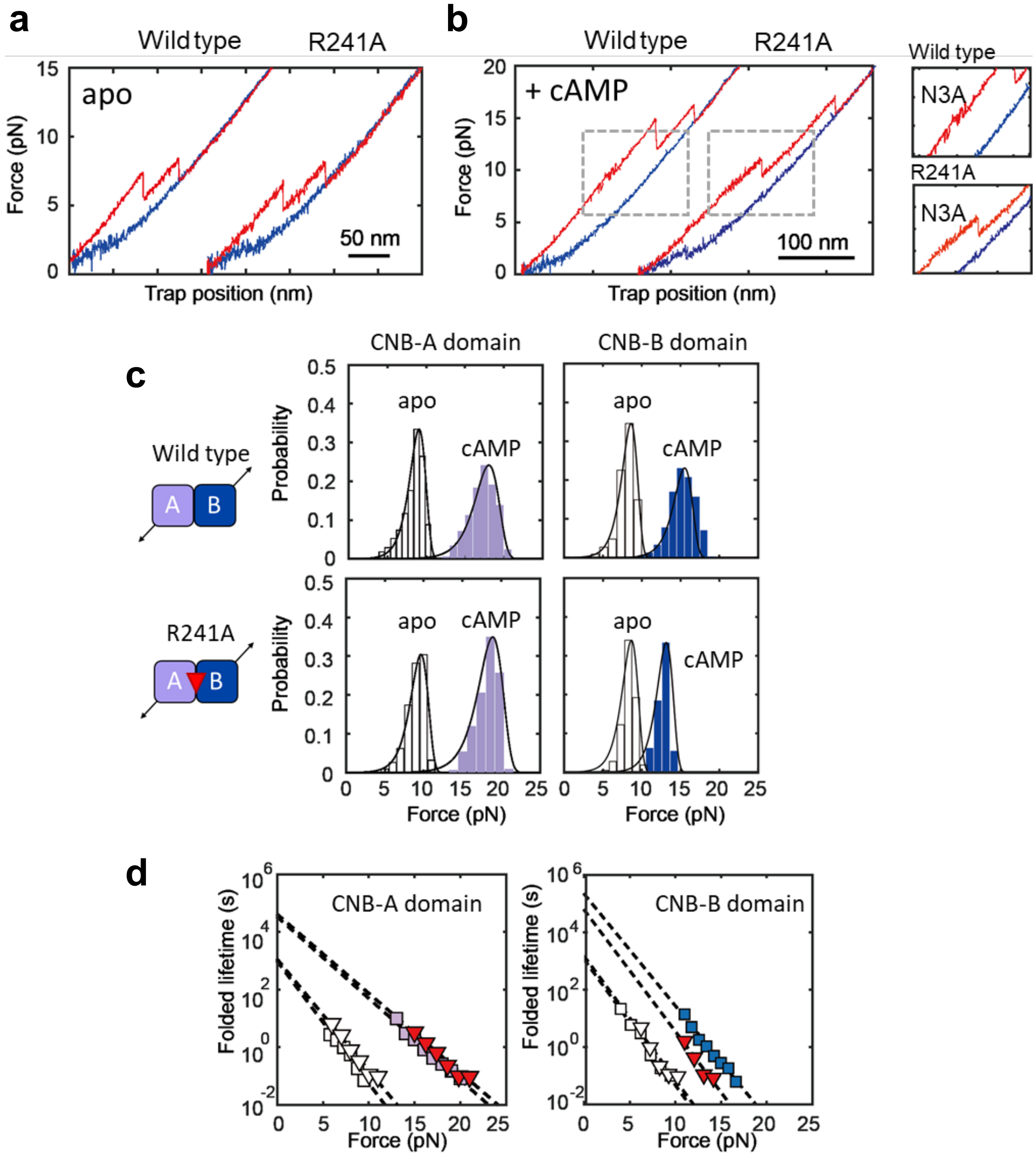

Supplementary Figure 6 | Comparison of type-III (S110C/S376C) wild type and R241A mutant. a, Force-extension curves of type-III constructs (S110C/S376C) for wild type and the R241A mutant regulatory subunits in apo state. **b,** Force-extension curves of type-III constructs (S110C/S376C) for wild type and the R241A mutant regulatory subunits obtained with cAMP (left). Zoomed-in trajectories showing the first reversible transition corresponding to the N3A motif in wild type and R241A (right). **c,** Unfolding force probability distributions for each CNB domain corresponding to wild type and R241A. The unfilled bar represents the apo state while filled bars represent cAMP-bound data. **d,** Force-dependent folded state lifetimes for each CNB domain in the apo state (empty symbols) and cAMP-bound conformation (colored symbols) for wild type (squares) and R241A (triangles).

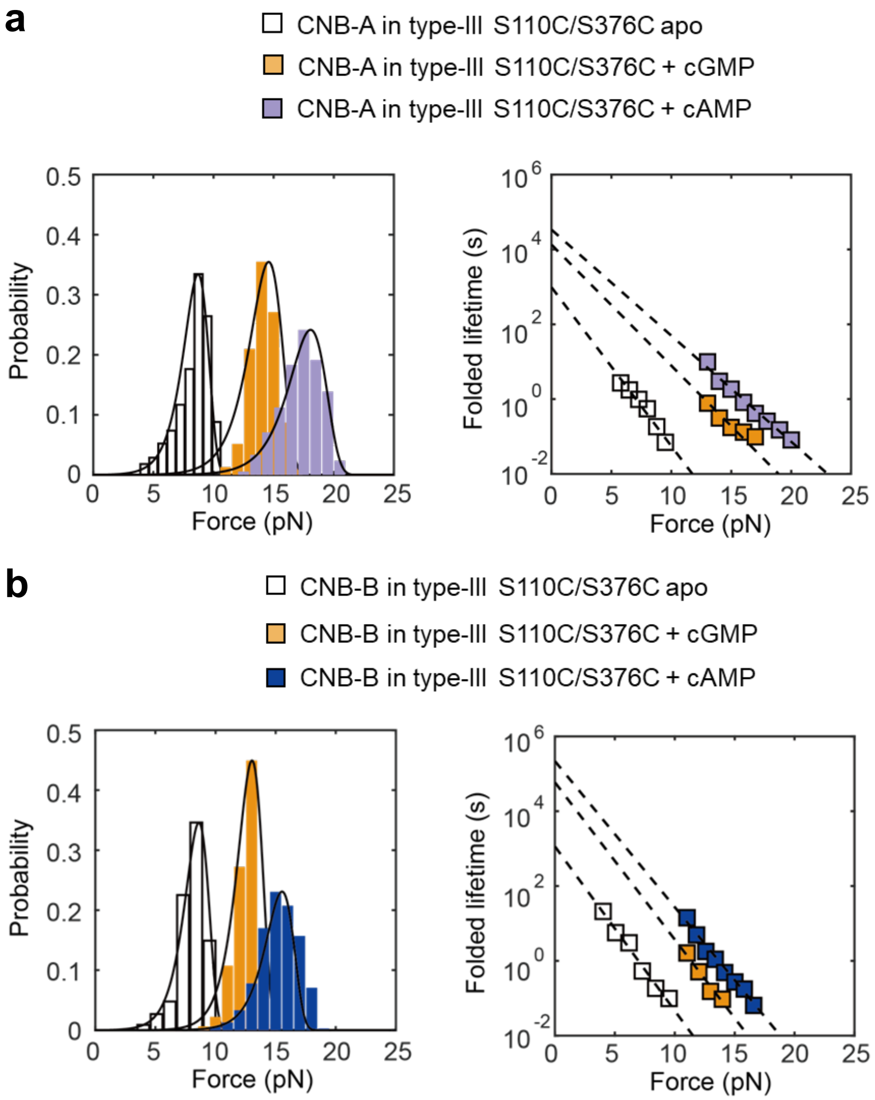

Supplementary Figure 7 | Comparison of unfolding force probability distributions (left) and corresponding force-dependent folded state lifetimes (right) for the type-III construct in apo, cGMP-, and cAMP-bound states. a, CNB-A domain and **b,** CNB-B in the regulatory subunit (type-III construct, S110C/S376C) in the apo state (empty symbols) or bound to cGMP (orange) or cAMP (purple and blue).
