# Supplementary material for: Activation of PKA via asymmetric allosteric coupling of structurally conserved cyclic nucleotide binding domains
Source: Nat Commun. 2019 Sep 4;10:3984. doi: 10.1038/s41467-019-11930-2 (PMC6726620; doi:10.1038/s41467-019-11930-2)
Supplement: Supplementary file 3 — Description of Additional Supplementary Files [file 41467_2019_11930_MOESM3_ESM.pdf]

## Description of Additional Supplementary Files

**File name:** Supplementary Movie 1

**Description:** Steered molecular dynamic simulation for the mechanical unfolding of the wild type PKA regulatory subunit bound to cAMP. The N3A motif is rendered in yellow, the CNB-A domain in magenta, and the CNB-B domain in blue. The protein is pulled from the C-terminus (red sphere) with a fix position in the N-terminus (blue sphere). The cAMP molecules are rendered as sticks.

**File name:** Supplementary Movie 2

**Description:** Steered molecular dynamic simulation for the mechanical unfolding of the wild type PKA regulatory subunit in the apo state. The N3A motif is rendered in yellow, the CNB-A domain in magenta, and the CNB-B domain in blue. The protein is pulled from the C-terminus (red sphere) with a fix position in the N-terminus (blue sphere).

**File name:** Supplementary Movie 3

**Description:** Steered molecular dynamic simulation for the unfolding of the PKA regulatory subunit harboring the mutation R241A (green sticks) bound to cAMP. The N3A motif is rendered in yellow color, the CNB-A domain in magenta, and the CNB-B domain in blue. The protein is pulled from the C-terminus (red sphere) with a fix position in the N-terminus (blue sphere). The cAMP molecules are rendered as sticks.
