# Supplementary material for: Activation of PKA via asymmetric allosteric coupling of structurally conserved cyclic nucleotide binding domains
Source: Nat Commun. 2019 Sep 4;10:3984. doi: 10.1038/s41467-019-11930-2 (PMC6726620; doi:10.1038/s41467-019-11930-2)
Supplement: Supplementary file 7 — Reporting Summary [file 41467_2019_11930_MOESM7_ESM.pdf]

nature research

Corresponding author(s): Rodrigo Maillard

Last updated by author(s): Apr 17, 2019

Reporting Summary

Nature Research wishes to improve the reproducibility of the work that we publish. This form provides structure for consistency and transparency in reporting. For further information on Nature Research policies, see Authors & Referees and the Editorial Policy Checklist.

Statistics

For all statistical analyses, confirm that the following items are present in the figure legend, table legend, main text, or Methods section.

n/a	Confirmed	
☐	☒	The exact sample size (n) for each experimental group/condition, given as a discrete number and unit of measurement
☐	☒	A statement on whether measurements were taken from distinct samples or whether the same sample was measured repeatedly
☐	☒	The statistical test(s) used AND whether they are one- or two-sided<br>*Only common tests should be described solely by name; describe more complex techniques in the Methods section.*
☒	☐	A description of all covariates tested
☒	☐	A description of any assumptions or corrections, such as tests of normality and adjustment for multiple comparisons
☐	☒	A full description of the statistical parameters including central tendency (e.g. means) or other basic estimates (e.g. regression coefficient) AND variation (e.g. standard deviation) or associated estimates of uncertainty (e.g. confidence intervals)
☐	☒	For null hypothesis testing, the test statistic (e.g. F, t, r) with confidence intervals, effect sizes, degrees of freedom and P value noted<br>*Give P values as exact values whenever suitable.*
☐	☒	For Bayesian analysis, information on the choice of priors and Markov chain Monte Carlo settings
☒	☐	For hierarchical and complex designs, identification of the appropriate level for tests and full reporting of outcomes
☒	☐	Estimates of effect sizes (e.g. Cohen's d, Pearson's r), indicating how they were calculated

Our web collection on statistics for biologists contains articles on many of the points above.

Software and code

Policy information about availability of computer code

Data collection	We use a custom made labview based software to collect single molecule optical tweezers data. The software is available upon request or found here: http://tweezerslab.unipr.it/cgi-bin/mt/software.pl/Search
Data analysis	We use Matlab-based codes to do the following analyses: 1) Determine unfolding or refolding forces and the associated change in extension of all target protein constructs. 2) Transform the unfolding and refolding force histograms into force-dependent lifetimes following the methods developed by Dudko and co-workers in PNAS 2008. 3) We use built-in non-linear square analysis (curve fitting tool in Matlab) to fit force dependent lifetimes and extract lifetimes at zero force and distances to the transition state. This allows us to determine the error of the fit. (4) We ran Monte Carlo simulations using Matlab. Description of the MC simulation is in the supporting information section of the manuscript.<br>We also use PyFolding to do global analysis of ligand binding data. PyFolding is available here: https://github.com/quantumjot/PyFolding

For manuscripts utilizing custom algorithms or software that are central to the research but not yet described in published literature, software must be made available to editors/reviewers. We strongly encourage code deposition in a community repository (e.g. GitHub). See the Nature Research guidelines for submitting code & software for further information.

Data

Policy information about availability of data

All manuscripts must include a data availability statement. This statement should provide the following information, where applicable:

- Accession codes, unique identifiers, or web links for publicly available datasets
- A list of figures that have associated raw data
- A description of any restrictions on data availability

The raw data that support the findings of this study are available from the corresponding author (R.M.) upon reasonable request.

Field-specific reporting

Please select the one below that is the best fit for your research. If you are not sure, read the appropriate sections before making your selection.

☒ Life sciences ☐ Behavioural & social sciences ☐ Ecological, evolutionary & environmental sciences

For a reference copy of the document with all sections, see nature.com/documents/nr-reporting-summary-flat.pdf

Life sciences study design

All studies must disclose on these points even when the disclosure is negative.

Sample size	All the distributions of single molecule data included between 200 and 1500 data points (described in Tables).
Data exclusions	Single molecule unfolding or refolding data outputs force and changes in extension. We excluded data that showed more than +/- 2 standard deviation of the mean of changes in extension. These exclusion limits represented less than 1% of the total data collected.
Replication	For a specific protein construct at a specific experimental condition, we collected single molecule data of at least 5 and up to 10 different molecules.
Randomization	This is not relevant to the study. Molecules corresponding to the same protein are subjected to optical tweezers under different conditions.
Blinding	This is not relevant to the study. The optical measurements are recorded automatically and cannot be biased by the investigator.

Reporting for specific materials, systems and methods

We require information from authors about some types of materials, experimental systems and methods used in many studies. Here, indicate whether each material, system or method listed is relevant to your study. If you are not sure if a list item applies to your research, read the appropriate section before selecting a response.

Materials & experimental systems

n/a	Involved in the study	
☒	☐	Antibodies
☒	☐	Eukaryotic cell lines
☒	☐	Palaeontology
☒	☐	Animals and other organisms
☒	☐	Human research participants
☒	☐	Clinical data

Methods

n/a	Involved in the study	
☒	☐	ChIP-seq
☒	☐	Flow cytometry
☒	☐	MRI-based neuroimaging
